# Investigating the genetic underpinnings of early-life irritability

**DOI:** 10.1038/tp.2017.212

**Published:** 2017-09-26

**Authors:** L Riglin, O Eyre, M Cooper, S Collishaw, J Martin, K Langley, E Leibenluft, A Stringaris, A K Thapar, B Maughan, M C O'Donovan, A Thapar

**Affiliations:** 1 MRC Centre for Neuropsychiatric Genetics and Genomics, Division of Psychological Medicine and Clinical Neurosciences, Cardiff University, Cardiff, Wales, UK; 2 Department of Medical Epidemiology and Biostatistics, Karolinska Institutet, Stockholm, Sweden; 3 School of Psychology, Cardiff University, Cardiff, Wales, UK; 4 Emotion and Development Branch, National Institute of Mental Health, Bethesda, MD, USA; 5 Social, Genetic and Developmental Psychiatry Centre, Institute of Psychiatry, Psychology and Neuroscience, King’s College London, London, UK

## Abstract

Severe irritability is one of the commonest reasons prompting referral to mental health services. It is frequently seen in neurodevelopmental disorders that manifest early in development, especially attention-deficit/hyperactivity disorder (ADHD). However, irritability can also be conceptualized as a mood problem because of its links with anxiety/depressive disorders; notably DSM-5 currently classifies severe, childhood-onset irritability as a mood disorder. Investigations into the genetic nature of irritability are lacking although twin studies suggest it shares genetic risks with both ADHD and depression. We investigated the genetic underpinnings of irritability using a molecular genetic approach, testing the hypothesis that early irritability (in childhood/adolescence) is associated with genetic risk for ADHD, as indexed by polygenic risk scores (PRS). As a secondary aim we investigated associations between irritability and PRS for major depressive disorder (MDD). Three UK samples were utilized: two longitudinal population-based cohorts with irritability data from childhood (7 years) to adolescence (15–16 years), and one ADHD patient sample (6–18 years). Irritability was defined using parent reports. PRS were derived from large genome-wide association meta-analyses. We observed associations between ADHD PRS and early irritability in our clinical ADHD sample and one of the population samples. This suggests that early irritability traits share genetic risk with ADHD in the general population and are a marker of higher genetic loading in individuals with an ADHD diagnosis. Associations with MDD PRS were not observed. This suggests that early-onset irritability could be conceptualized as a neurodevelopmental difficulty, behaving more like disorders such as ADHD than mood disorders.

## Introduction

Severe irritability has long been recognized as a common accompanying difficulty to neurodevelopmental disorders that manifest early in development. Irritability is commonly defined as a heightened propensity to react with anger, grouchiness, or tantrums relative to peers and is particularly common in attention-deficit/hyperactivity disorder (ADHD).^1, 2, 3, 4^ Recent estimates suggest it is present in around 25–45% of children with ADHD, when defined broadly as emotion dysregulation (which can include an inability to control other emotions, such as sadness).^1^ As well as being common, emerging evidence suggests that irritability—as well as broader constructs which include irritability (for example, emotion dysregulation, behavioral problems)—appears to index clinical severity and is associated with adverse outcomes in individuals with ADHD.^4, 5^ It is currently unclear how irritability should be conceptualized, particularly because it is a trans-diagnostic construct. Within DSM-5, irritability is included as a feature of a number of different diagnostic categories. Severe chronic childhood irritability has recently been categorized by the new DSM-5 diagnosis of disruptive mood dysregulation disorder (DMDD) and classed as a mood disorder because of links with later depression.^6^ However, childhood irritability behaves similarly to ADHD (and other neurodevelopmental disorders) in that onset is early in development and levels tend to decline from childhood to adolescence,^7, 8^ raising the possibility that earlier-onset irritability behaves more like a neurodevelopmental difficulty such as ADHD, rather than a mood disorder.

Irritability is one of the most common reasons prompting referral to mental health services.^9^ Yet, despite the clinical importance of irritability, there is a limited understanding of its genetic architecture. Twin studies suggest that irritability has a heritability estimate of 30–40%.^10^ To date, one twin study has reported significant genetic overlap between emotional dysregulation (a construct closely related to irritability) and ADHD symptoms,^11^ and family studies also suggest that emotional lability is elevated in family members of individuals with ADHD.^12, 13^ However other twin study findings suggest that irritability shares genetic liability with depression.^14^ Molecular genetic investigations could help clarify the genetic underpinnings of irritability but currently investigations at a molecular level are lacking.

Large genome-wide association studies of patients and controls can be used (as discovery samples) to derive individual composite genetic risk scores (PRS—polygenic risk scores) that serve as an index of genetic liability for the disorder in an independent (target) sample.^15^ Given that irritability is so strongly associated with ADHD, our primary aim was to test the hypothesis that early irritability would be associated with ADHD PRS. We set out to test this hypothesis in two longitudinal population-based cohorts with irritability measured in childhood and adolescence, and in a clinic-based sample of young people with ADHD. As a secondary aim, given the new classification of chronic childhood irritability as a mood disorder in DSM-5, we also investigated associations between irritability and PRS for major depressive disorder (MDD).

## Materials and methods

### Samples

Three UK samples were included in the study: two population based cohorts (the Avon Longitudinal Study of Parents and Children, ALSPAC,^16, 17^ and the National Child Development Study, NCDS)^18^ and one clinical sample (the Study of ADHD, Genes and Environment, SAGE)).^19^

The Avon Longitudinal Study of Parents and Children (ALSPAC) is a well-established prospective, longitudinal birth cohort study. The enrolled core sample consisted of 14 541 mothers living in Avon, England, who had expected delivery dates of between April 1, 1991 and December 31, 1992. Of these pregnancies 13 988 children were alive at 1 year. Where families included twins, we included the oldest sibling. When the oldest children were ~7 years of age, the initial sample was augmented with eligible cases who had failed to join the study originally, resulting in an additional 713 children being enrolled. There was a total of 14 701 children alive at 1 year of age. In total 9912 ALSPAC children were genotyped, of whom 8365 passed quality control. Full details of the study, measures and sample can be found elsewhere.^16, 17^ The study website contains details of all the data that is available through a fully searchable data dictionary (http://www.bris.ac.uk/alspac/researchers/data-access/data-dictionary). Ethical approval for the study was obtained from the ALSPAC Ethics and Law Committee and the Local Research Ethics Committees.

The National Child Development Study (NCDS) is another well-established prospective, UK birth cohort. The study recruited 18 558 children from England, Wales and Scotland born during one week in 1958. Genotype data collected at age 44 years were available for 5257 individuals following quality control. Full details of the study are described elsewhere.^18^ Ethical approval for the biomedical survey from which genetic data were available was obtained from the South East Multicentre Research Ethics Committee.

The Study of ADHD, Genes and Environment (SAGE) is a clinical sample of children with ADHD recruited from UK child psychiatry and pediatric clinics between 2007 and 2011. The sample consisted of 696 participants (84% male) aged 6–18 years (mean=10.9, s.d.=2.99) with a clinical diagnosis of ADHD that was confirmed by research diagnostic interviews. Genotype data were available for 569 individuals following quality control. Full details of the study are described elsewhere.^19^ Ethical approval for the study was obtained from the Wales Multicentre Research Ethics Committee.

### Polygenic risk scores

PRS were generated as the weighted mean number of disorder risk alleles in approximate linkage equilibrium using standard procedures.^20^ ADHD and MDD risk alleles were identified from Psychiatric Genomics Consortium (PGC) analysis of case-control GWAS. For testing ADHD PRS in ALSPAC, 9 ADHD studies were included in the ADHD discovery data set (5621 cases and 13 589 controls).^21, 22, 23^ As the ADHD discovery GWAS included data from NCDS (controls) and SAGE (cases), meta-analyses (using the standard error scheme in METAL) excluding these samples were performed to identify risk alleles for the purpose of generating PRS in NCDS and SAGE (4817 cases and 6510 controls for NCDS; 4980 cases and 11 837 controls for SAGE). For all three samples, eight studies were included in the MDD discovery data set (9240 cases and 9519 controls).^24^ Primary analyses defined risk alleles as those associated at *p*-threshold<0.5 (previously reported to maximally capture phenotypic variance for ADHD and MDD); associations across a range of *p*-thresholds are shown in Supplementary Figure 1. Further details of the methods for generating the PRS have been described elsewhere^25^ and are given in Supplementary Material. Genotyping details are given in Supplementary Material.

### Irritability

Irritability was defined using available parent-reported measures in all three samples. Details of the specific items, response scale and requirements for a participant to be classed as irritable in each sample are described in the text below and summarized in Table 1. The irritability items were selected on the basis of previous research^26, 27, 28^ and counted as present if they occurred frequently in the young person (see Table 1 for details). We generated irritability constructs that were similar but not identical across each sample given that different measures were available in each data set.

In ALSPAC, irritability was assessed at three time-points using the Development and Well-Being Assessment (DAWBA)^29^ (a structured research diagnostic interview) at ages 7 years 7 months, 10 years 8 months and 15 years 6 months. Individuals were categorized as irritable if they were rated as having at least one of three symptoms (severe temper tantrums, touchy and easily annoyed, angry and resentful) ‘a lot more than others’.

In NCDS, irritability data were available from a single item from the abbreviated Rutter A scale^30^ at three time points—ages 7, 11 and 16 years. Individuals were categorized as irritable if they were rated as being ‘frequently irritable, quick to fly off the handle’.

In the clinical sample of participants with ADHD (SAGE), irritability was assessed at one time point (age range 6–18 years) using the Child and Adolescent Psychiatric Assessment (CAPA)^31^ (a semi-structured research diagnostic interview). Individuals were categorized as irritable if they were rated as having at least one of three symptoms (temper tantrums, touchy or easily annoyed or angry and resentful). These had to occur frequently over the past three months (at least four times per week for touchy/easily annoyed or angry/resentful, or three times per week for temper tantrums), uncontrollably, and interfere with at least two activities. For sensitivity analyses a continuous score (possible range 0–3) was also generated.

### ADHD symptoms

ADHD symptoms were measured using the parent-reported DAWBA in ALSPAC (corresponding to the 18 DSM-IV symptoms, possible range 0–36), the Rutter A scale in NCDS (two items: ‘is squirmy or fidgety’ and ‘has difficulty in settling to anything for more than a few moments’/‘very restless, has difficulty staying seated for long’, possible range 0–4) and the CAPA in SAGE (18 DSM symptoms, possible range 0–18).

### Analyses

We first examined irritability in each of the samples: specifically, how common it was, the prevalence at different ages, and associations with sex and total ADHD symptom scores.

The primary analyses tested for associations between ADHD PRS and irritability using logistic regressions in SPSS. Sex and 10 population stratification principal components were included as covariates in all PRS analyses. Proportion of variance explained was calculated as the difference between the Nagelkerke pseudo-R-square in the model including PRS compared with the null model that did not include PRS (Δ*R*^2^). Developmental differences were assessed in the longitudinal population samples, by comparing genetic risk associations with irritability in childhood and adolescence. In the clinical ADHD sample, measures were available at only one time point; thus, sensitivity analyses were run splitting the sample into two age groups (<12 years (*N*=408) and ⩾12 years (*N*=288)). In all three samples, effects of age were investigated by testing for a PRS-by-age interaction (a random effects model was included for the population samples where irritability was assessed at multiple time points).

## Results

Table 2 shows descriptive information about irritability in each of the samples.

In ALSPAC, around 4% of the sample met the criteria for irritability at each of the three time points (ages 7, 10 and 15 years). More boys than girls met the criteria in childhood (ages 7 and 10) but not in adolescence (age 15)—there was a sex-by-age interaction (z=−4.72, *P*<0.001) whereby irritability decreased from ages 7 to 15 in boys and increased in girls (z=−1.76, *P*=0.078 and z=4.68, *P*<0.001 respectively).

In NCDS, between 9 and 13% of the sample met the criteria for irritability at each of the three time points (age 7, 11 and 16). Again, more boys met the criteria in childhood (age 11), while more girls met the criteria in adolescence (age 16), with a sex-by-age interaction (*z*=−5.31, *P*<0.001) whereby irritability decreased from age 7 to 16 in boys and increased in girls (*z*=−5.26, *P*<0.001 and *z*=2.12, *P*=0.034 respectively).

Thus, despite different prevalence rates (and measures) of irritability in the two population samples, both showed similar developmental trends and sex differences.

In SAGE, the patient sample, 91% of the sample met criteria for irritability, with no sex difference in prevalence although irritability was more common in the ‘younger’ (⩽11 years) than ‘older’ (⩾12 years) subsamples (93.5% and 87.0% respectively, χ^2^(1)=8.36, *P*=0.004).

Irritability was consistently associated with ADHD total symptom scores at all ages in all samples (Table 2).

### Association between irritability and ADHD PRS

Associations between ADHD PRS and irritability are given in Table 3. Within the population-based samples, ADHD PRS were associated with irritability in ALSPAC at all ages. In NCDS, ADHD PRS were not associated with irritability (*P*<0.1 at age 7 only). Within the ADHD clinical sample (SAGE), ADHD PRS were associated with irritability. Because of the high prevalence of irritability in the clinical sample, a continuous measure of irritability was also examined. The pattern of association was similar, but non-significant (β=0.077, *P*=0.073, see Supplementary Table 1).

Sensitivity analyses in the clinical ADHD sample indicated no clear age effects (younger subsample OR=1.48 (0.92–2.39), *P*=0.110; older subsample OR=1.53 (0.99–2.36), *P*=0.053) and there was no evidence of any interaction between ADHD PRS score and either age or sex at *P*<0.05 in any of the samples.

### Association between irritability and MDD PRS

Associations between MDD PRS and irritability are shown in Table 4. MDD PRS were not associated with irritability in either of the population samples at any age, or in the ADHD clinical sample (SAGE, including when using a continuous score, see Supplementary Table 1). Sensitivity analyses in the clinical ADHD sample indicated no clear age effects (younger subsample OR=0.84 (0.54–1.32), *P*=0.448; older subsample OR=1.19 (0.77–1.81), *P*=0.435). The only evidence of an interaction between MDD PRS score and age across samples was a stronger association at age 7 than age 10 in ALSPAC (*z*=−2.04, *P*=0.041); there was no evidence for an interaction between MDD PRS and sex at *P*<0.05 in any of the samples.

## Discussion

This study aimed to investigate the genetic underpinnings of early irritability, by testing the hypothesis that it would be associated with ADHD genetic liability, as indexed by polygenic risk scores for clinical ADHD. We observed associations between ADHD PRS and early irritability in a population-based cohort and in an ADHD clinical sample. These findings are in keeping with those from a previous twin study and suggest modest genetic overlap between ADHD and irritability at a molecular (common variant) level.^21, 22, 23^ The results from the patient ADHD sample further suggest that irritability is a clinical marker of genetic loading in this group.

There are several reasons why ADHD PRS might be associated with irritability. One possibility is that it is a core feature of ADHD: irritability was historically included as an associated feature of ADHD and it remains a commonly co-occurring symptom.^1, 32^ It also appears to show similar epidemiological patterns to ADHD and many other DSM-5 neurodevelopmental disorders—symptom levels have been reported to be highest in childhood and to reduce with age,^8^ a pattern we observed in males. We also found a male preponderance for irritability in our population samples, at least in childhood,^33^ although sex differences in irritability have not been consistent in previous work (see below). There is also some evidence that effective ADHD treatment with stimulant medication improves irritability in children with ADHD.^34^ However, symptoms of irritability are not prominent in all children with ADHD^1^ (although they are very common in our clinical sample), suggesting that irritability might provide additional information regarding severity of disorder and genetic loading within those with ADHD. Notably, associations between ADHD PRS and irritability were similar after taking into account ADHD (and Conduct Disorder) symptom levels in our clinical sample, suggesting these are not entirely the same constructs (Supplementary Material).

Another possibility is that ADHD common genetic risk variants have pleiotropic effects on ADHD and early irritability in the population. Studies have already shown that ADHD polygenic risk scores are associated with other features that commonly accompany ADHD, such as lower IQ, working memory and conduct disorder,^35, 36^ as well as ADHD trait levels in the general population.^37, 38^ This is the first study that we are aware of that has shown an association with irritability, thereby addressing a gap in knowledge of the genetic architecture of early irritability. Twin studies highlight that psychopathology co-occurs as a result of shared genetic risks^39^ and molecular genetic studies concur in showing that ADHD genetic risk variants impact upon a wide range of early neurodevelopmental and behavioral traits. It is possible that ADHD PRS could have non-specific effects on multiple psychiatric traits in early life, although findings do suggest there could be some specificity in the pattern of effects.^36^

Although our study suggests that early irritability is associated with ADHD genetic risk scores, it is also important to note that previous longitudinal studies have found associations between early irritability and later emotional/mood disorders in general population samples:^10, 14^ links which appear to be partially genetically mediated.^14, 40^ Such findings have suggested that childhood irritability is an early manifestation of mood problems. Indeed severe childhood-onset irritability (DMDD) is classified as a mood disorder in the DSM-5. The DSM-5 classification of childhood-onset irritability as a mood disorder would predict an association between early irritability and MDD PRS, which we did not find. However, interestingly, in both of our population-based cohorts we found developmental differences in the pattern of prevalence rates of irritability for males and females; the prevalence decreased from childhood to adolescence in males (a pattern typical of neurodevelopmental disorders) but increased for females (a pattern typical of mood disorders). Although previous studies have not consistently found sex differences in irritability,^8, 41, 42, 43, 44, 45^ the age range and type of sample (e.g. clinical vs population) in which irritability has been measured has varied across studies, which may in part explain the differing findings. We suggest that the manifestation of irritability might represent different underlying problems depending on age and sex; that is, it could be more like ADHD in childhood and more like mood disorder later in development. The findings from this study suggest that future studies need to adopt a developmental approach in defining and investigating irritability, for example by considering age of onset, even though this is not an explicit requirement in R-DoC.^46, 47^

Secondary analyses found no association between irritability and PRS for MDD in our samples (see Supplementary Table 2). However, these findings should be interpreted cautiously. Despite similar GWAS discovery sample sizes (*N*=18 759 for MDD; *N*=19 210 for ADHD), MDD PRS will be underpowered compared with ADHD PRS for several reasons, including that MDD is more common and less heritable than ADHD.^48^ Another difference worth noting is that while the ADHD PRS were generated based on a GWAS on children with ADHD, the MDD PRS were generated based on a GWAS of adult MDD. Indeed, there is emerging evidence that the genetic architecture of MDD may differ by age-at-onset^48^ and MDD PRS derived from a GWAS of younger patients with MDD may have resulted in stronger associations with childhood irritability. Nevertheless, if childhood irritability is an early manifestation of later, adult, mood problems, an association between childhood irritability and adult-MDD PRS would be predicted. Thus, while we can likely exclude large effect sizes of MDD PRS on early irritability, we cannot rule out an association with genetic liability for depression, especially given findings from twin studies.^14, 40^ Large scale international molecular genetic studies show genetic overlap between ADHD and depression,^49, 50, 51^ and these disorders co-occur more often than would be expected by chance;^52^ as a result, it is possible that irritability is a common factor between the two.

Another explanation is that early irritability is neurodevelopmental in nature (that is, manifest early and more common in males), but its links with later depression are mediated via gene-environment correlation (for example via eliciting adverse social stressors such as peer rejection). Such mechanisms would be incorporated into estimates of shared genetic effects in twin studies. Research into whether irritability mediates the association between ADHD and later depression is ongoing.^4, 53^ We encourage future work investigating irritability across different developmental stages, across sex and within different disorders to help identify similarities, as well as differences in presentation, associated characteristics and treatment response. The findings from this study suggest that while irritability is a trans-diagnostic construct, future studies need to adopt a developmental approach in defining and investigating it.

Although there are a number of strengths to this study—in particular the use of three independent samples—there are also a number of limitations that should be noted. First, measurement of irritability varied across the three samples. In ALSPAC and SAGE we used three items, which have previously been used to measure irritability (informed by factor analyses),^3, 28, 54, 55^ although in ALSPAC parents were asked about irritability compared with others, while in SAGE parents were asked about the presence/absence of irritability. In NCDS we were limited to one irritable item, which asked about the frequency of irritability, and was more frequently endorsed than in ALSPAC (for example, 10.4% compared with 3.9%, at age 7) and thus likely represents a less severe presentation of irritability. Measurement differences may have influenced observed associations. Despite different measures we did observe associations between ADHD PRS and irritability in both the ADHD clinical sample and the other population sample (ALSPAC). In fact, these consistent findings across samples and varying instruments and definitions of irritability increase confidence about their robustness. It is also worth noting that the level of irritability we are measuring in all three samples (at least one frequent irritable symptom), is not equivalent to the chronic severe irritability that characterizes the new diagnosis of DMDD, which has been researched by others.

Second, ALSPAC and NCDS are longitudinal birth cohort studies that show non-random attrition. Specifically, individuals with lower ADHD PRS and lower levels of irritability are more likely to have remained in the studies.^56, 57^ This may have been particularly problematic in NCDS where DNA was collected at age 44 years (for example, ADHD, especially in those also with behavioral problems including irritability, is associated with premature death).^5^ Such bias may have resulted in an underestimation of the association between ADHD PRS and irritability, particularly in adolescence when there was more attrition.

Finally, the PRS were based on an available discovery GWAS and only explain a small proportion of phenotypic variance due to current discovery sample sizes,^58^ (with SNP heritability estimated to be roughly 0.2–0.3 for ADHD and MDD^20^). Nevertheless, they are useful biological indicators of disease risk,^20^ that can aid the investigation of the genetic architecture of phenotypes in different samples. Our effect sizes are in line with other work using similar approaches.^36, 59^

In conclusion, this study suggests that irritability, when manifest during childhood and adolescence in the general population and in patients with ADHD, is associated with ADHD genetic liability as indexed by PRS. This finding, coupled with observations that irritability tends to decline from childhood to adolescence, suggests early irritability is similar to ADHD and, when early in onset, may be better conceptualized as a neurodevelopmental difficulty rather than a mood disorder-related problem. Further work is needed to better understand the developmental nature of irritability and its links with psychiatric disorders.^14^

## Figures and Tables

**Table 1:** Measures of irritability

*Sample*	*Assessment*	*Irritability item(s)*	*Response scale*	*Requirement to code as irritable*
ALSPAC	Development and Well-Being Assessment (DAWBA)	Severe temper tantrums Touchy and easily annoyed Angry and resentful	No more than others A little more than others A lot more than others	At least one irritable item rated as happening a lot more than others in the last 6 months compared with other children of the same age
NCDS	Abbreviated Rutter A scale	Is irritable, quick to fly off the handle	Never/doesn’t apply^a^ Sometimes/applies somewhat^a^ Frequently/certainly applies^a^	Irritable item rated as happening frequently/certainly applies
SAGE	Child and Adolescent Psychiatric Assessment (CAPA)	Temper tantrums^b^ Touchy or easily annoyed^c^ Angry or resentful^d^	Symptom absent Symptom present	At least one irritable item rated as occurring at least 4 times per week (or 3 times per week for temper tantrums), uncontrollable and interfering with at least 2 activities over past 3 months

Abbreviations: ALSPAC, The Avon Longitudinal Study of Parents and Children; NCDS, The National Child Development Study; SAGE, The Study of ADHD, Genes and Environment.

a At age 7 and 11, response scale was never, sometimes and frequently; at age 16, response scale was doesn’t apply, applies somewhat and certainly applies.

b Temper tantrums defined as ‘discrete episodes of excessive temper, frustration or upset, manifested by shouting, crying or stamping, and involving violence or attempts at damage directed against people or property’.

c Touchy or easily annoyed defined as ‘generally more prone to feelings of anger, bad temper, short temper, resentment, sulking or annoyance, under minor provocation than most children.

d Angry or resentful defined as ‘generally more prone to manifestations of anger or resentment (such as snappiness, shouting, quarreling or sulking) under minor provocation, than most children.

**Table 2:** Irritability descriptive statistics and associations with ADHD symptom levels and sex

*(a) Population sample: ALSPAC*	*Age 7*	*Age 10*	*Age 15*
Sample N	7963	7558	4602
Frequency	3.9%	4.0%	4.2%
Frequency in males	5.2%	5.1%	3.8%
Frequency in females	2.5%	2.9%	4.5%
Sex difference (χ^2^(1))	40.20, *P*<0.001	23.72, *P*<0.001	1.26, *P*=0.262
ADHD symptoms (OR)	1.17 (1.16–1.19) *P*<0.001	1.18 (1.17–1.20) *P*<0.001	1.18 (1.16–1.20) *P*<0.001

*(b) Population sample: NCDS*	*Age 7*	*Age 11*	*Age 16*
Sample N	8201	8011	7063
Frequency	10.4%	13.1%	9.4%
Frequency in males	10.9%	14.1%	7.7%
Frequency in females	9.9%	12.0%	11.1%
Sex difference (χ^2^(1))	1.97, *P*=0.160	7.78, *P*=0.005	24.47, *P*<0.001
ADHD symptoms (OR)	1.90 (1.80–2.02) *P*<0.001	1.79 (1.70–1.89) *P*<0.001	1.78 (1.67–1.90) *P*<0.001

*(c) ADHD sample: SAGE*		*(Age 6–18)*	
Sample N		678	
Frequency		90.9%	
Frequency in males		89.0%	
Frequency in females		91.2%	
Sex difference (χ^2^(1))		0.54, *P*=0.461	
ADHD symptoms (OR)		1.25 (1.16–1.35) *P*<0.001	

Abbreviations: ADHD, attention-deficit/hyperactivity disorder; ALSPAC, The Avon Longitudinal Study of Parents and Children; NCDS, The National Child Development Study; SAGE, The Study of ADHD, Genes and Environment.

*N*=sample with irritability data.

**Table 3:** Associations between ADHD polygenic risk scores and irritability

	*OR*	(*95% CI*)	p	ΔR^2^
*(a) Population sample: ALSPAC*				
Age 7 (*N*=5584)	1.20	(1.04–1.38)	0.010	0.005
Age 10 (*N*=5432)	1.22	(1.06–1.40)	0.007	0.005
Age 15 (*N*=3602)	1.20	(1.02–1.42)	0.031	0.004
				
*(b) Population sample: NCDS*				
Age 7 (*N*=4960)	1.09	(0.99–1.20)	0.085	0.002
Age 11 (*N*=4544)	1.06	(0.97–1.15)	0.220	0.001
Age 16 (*N*=4023)	1.02	(0.91–1.13)	0.779	0.0001
				
*(c) ADHD sample: SAGE*				
Age 6–18 (*N*=560)	1.37	(1.02–1.86)	0.039	0.017

Abbreviations: ADHD, attention-deficit/hyperactivity disorder; ALSPAC, The Avon Longitudinal Study of Parents and Children; NCDS, The National Child Development Study; SAGE, The Study of ADHD, Genes and Environment.

ADHD polygenic risk score using *p*-threshold <0.5. Analyses controlling for sex and 10 principle components.

**Table 4:** Associations between MDD polygenic risk scores and irritability

	*OR*	*(95% CI)*	P	ΔR^*2*^
*(a) Population sample: ALSPAC*				
Age 7 (*N*=5584)	1.09	(0.95–1.25)	0.239	0.001
Age 10 (*N*=5432)	0.91	(0.79–1.05)	0.184	0.001
Age 15 (*N*=3602)	1.02	(0.87–1.21)	0.785	0.0001
				
*(b) Population sample: NCDS*				
Age 7 (*N*=4690)	1.01	(0.92–1.12)	0.793	0.0001
Age 11 (*N*=4544)	1.07	(0.98–1.17)	0.153	0.001
Age 16 (*N*=4023)	0.97	(0.86–1.08)	0.536	0.0002
				
*(c) ADHD sample: SAGE*				
Age 6–18 (*N*=560)	1.05	(0.78–1.41)	0.763	0.0004

Abbreviations: ALSPAC, The Avon Longitudinal Study of Parents and Children; MDD, major depressive disorder; NCDS, The National Child Development Study; SAGE, The Study of ADHD, Genes and Environment.

MDD polygenic risk score using *p*-threshold <0.5. Analyses controlling for sex and 10 principle components.
