# Supplementary material for: Investigating the genetic underpinnings of early-life irritability
Source: Transl Psychiatry. 2017 Sep 26;7(9):e1241–. doi: 10.1038/tp.2017.212 (PMC5639253; doi:10.1038/tp.2017.212)
Supplement: Supplementary Materials [file tp2017212x1.docx]

**Supplementary Materials**

**ALSPAC**

In total 9 912 ALSPAC children were genotyped, of whom 8 365 passed quality control. Full genotyping details and individual exclusion criteria are described elsewhere (1, 2). Known autosomal variants were imputed with MACH 1.0.16 Markov Chain Haplotyping software (3, 4) using CEPH individuals from phase 2 of the HapMap project (HG18) as a reference set (release 22) resulting in a total N=2 543 887 SNPs. Dosage data were transformed from MACH output to PLINK format using fcGENE (5). After quality control exclusions (call rate <95%, MAF <1%, HWE P>5x10-7, R2≥.7) there were 1 813 169 autosomal SNPs. Individuals with genetic data who were alive at one year were included in this study (N=8 125).

**NCDS**

In total 5 514 NCDS participants were genotyped at age 44 years, of whom 5 257 passed quality control. Full genotyping details are described elsewhere (6, 7): roughly half of the individuals were genotyped on the Infinium HumanHap 550K v3 and half on the Illumina 1.2M (N=2 519 and 2 738 respectively). Individuals were excluded based on minimal/excess heterozygosity, incorrect gender assignment, cryptic relatedness, duplicate entries and being of non-European ancestry (detected using principal component analysis using the 1000 genomes dataset). 510 982 autosomal SNPs passed quality control exclusions (MAF <1%, call rate <95%, HWE p<5x10-7). Batch was included as a covariate in all analyses.

**SAGE**

In total 674 SAGE participants were genotyped, of whom 569 passed quality control. Roughly two-thirds of these were genotyped on the Illumina (San Diego) Human660W-Quad BeadChip (more details provided elsewhere; 8) and one third on the Illumina’s Infinium platform using the custom Institute of Psychological Medicine and Clinical Neurology chip (IPMCN, Cardiff University) (9) (N=354 and 215 respectively). Individuals were excluded based on minimal/excess heterozygosity, incorrect gender assignment, cryptic relatedness (where one of each pair of individuals related at least at the level of second cousins (pi-hat>0.05 was excluded), duplicate entries and being of non-European ancestry (assessed using principal components analysis). Only autosomal variants with MAF>0.01, call rate>0.99 that did not deviate from Hardy-Weinberg equilibrium at p<1×10-5 were included. Overlapping genotyped SNPs (N=124 776) that passed quality control exclusions in both samples were merged using PLINK. Batch was included as a covariate in all analyses.

**Population stratification**

The ALSPAC team used EIGENSTRAT principal components analysis to generate the top 100 components after the removal of known regions of long linkage disequilibrium in the data (3, 10). EIGENSTRAT analysis revealed no additional obvious population stratification and genome-wide analyses with other phenotypes indicated a low lambda.

We investigated possible population stratification in SAGE and NCDS. We first extracted the top 20 principal components, using --pca in PLINK, based on SNPs in relative linkage equilibrium (using --indep-pairwise 500 5 0.25 in PLINK to remove SNPs when pair-wise linkage disequilibrium was estimated to be greater than R2=.25, using windows of 500 SNPs, shifting 5 SNPs along at a time). We plotted a scree plot of the 20 principal components in descending order against the eigenvalues, to examine visually which components explained most of the variability in the genetic data. In NCDS there was a break whereby after the first 5 components, little additional variance was accounted for (see Supplementary Figure 2a). In SAGE there was no clear break in the scree plot, consistent with the sample predominantly being recruited from South Wales (see Supplementary Figure 2b).

For consistency across samples, we included the top ten principal components, as well as sex, as covariates. Batch was also included where appropriate (i.e. for NCDS and SAGE).

**Post-hoc analyses**

Association between ADHD PRS and irritability in the ADHD clinical sample (SAGE), adjusting for ADHD symptom severity: OR=1.48 (1.08-2.04) p=0.016, ΔR^2^=0.022.

Association between ADHD PRS and irritability in the ADHD clinical sample (SAGE), adjusting for conduct disorder symptom severity: OR=1.32 (0.98-1.80) p=0.072, ΔR^2^=0.012.

**Supplementary Table 1: Associations between polygenic risk scores (ADHD and MDD) and a continuous measure of irritability in SAGE sample**

|  | β | SE | p | ΔR^2^ |
| --- | --- | --- | --- | --- |
| ADHD PRS | 0.077 | 0.043 | 0.073 | 0.006 |
| MDD PRS | -0.039 | 0.042 | 0.354 | 0.001 |

SAGE = Study of ADHD, Genes and Environment. MDD= Major Depressive Disorder. PRS= polygenic risk score. Continuous measure of irritability: total score of 0-3 based on presence or absence of Child and Adolescent Psychiatric Assessment (CAPA) items “temper tantrums”, “touchy or easily annoyed” or “angry and resentful”. Analyses conducted in Mplus using a maximum likelihood parameter estimator (MLR) for which standard errors are robust to non-normality,^11^ controlling for sex and 10 principle components.

**Supplementary Figure 1**. Associations between polygenic risk scores and irritability, using a range of p-value thresholds from the discovery sample

| a) ADHD polygenic risk scores |
| --- |
|  |
| b) Major depressive disorder polygenic risk scores |
|  |

ALSPAC = The Avon Longitudinal Study of Parents and Children, NCDS = The National Child Development Study, SAGE = The Study of ADHD, Genes and Environment. OR per standard deviation increase in PRS, with 95% confidence intervals.

**Supplementary Figure 2**. Scree plot for the top 20 principal components for SAGE and NCDS.

| a) NCDS | b) SAGE |
| --- | --- |
|  |  |

NCDS = The National Child Development Study, SAGE = The Study of ADHD, Genes and Environment.

**Supplemental References**

1. Li Y, Willer CJ, Ding J, Scheet P, Abecasis GR. MaCH: using sequence and genotype data to estimate haplotypes and unobserved genotypes. *Genet Epidemiol*. 2010; **34**(8):816-34.
2. Li Y, Willer C, Sanna S, Abecasis G. Genotype imputation. *Annu Rev Genomics Hum Genet*. 2009; **10**:387.
3. Price AL, Patterson NJ, Plenge RM, Weinblatt ME, Shadick NA, Reich D. Principal components analysis corrects for stratification in genome-wide association studies. *Nat Genet*. 2006; **38**(8):904-9.
4. Price AL, Weale ME, Patterson N, Myers SR, Need AC, Shianna KV, et al. Long-range LD can confound genome scans in admixed populations*. Am J Hum Genet*. 2008; **83**(1):132-5.
5. Roshyara NR, Scholz M. fcGENE: A Versatile Tool for Processing and Transforming SNP Datasets. *PLoS One*. 2014; **9**(7):e97589. doi: 10.1371/journal.pone.0097589.
6. Barrett JC, Clayton DG, Concannon P, Akolkar B, Cooper JD, Erlich HA, et al. Genome-wide association study and meta-analysis find that over 40 loci affect risk of type 1 diabetes. *Nat Genet*. 2009; **41**(6):703-7.
7. Barrett JC, Lee JC, Lees CW, Prescott NJ, Anderson CA, Phillips A, et al. Genome-wide association study of ulcerative colitis identifies three new susceptibility loci, including the HNF4A region. *Nat Genet*. 2009; **41**(12):1330-4.
8. Stergiakouli E, Hamshere M, Holmans P, Langley K, Zaharieva I, Hawi Z, et al. Investigating the contribution of common genetic variants to the risk and pathogenesis of ADHD. *Am J Psychiat*. 2012; **169**(2):186-94.
9. Caseras X, Tansey K, Foley S, Linden D. Association between genetic risk scoring for schizophrenia and bipolar disorder with regional subcortical volumes. *Transl Psychiatry*. 2015; **5**(12):e692.
10. Price AL, Weale ME, Patterson N, Myers SR, Need AC, Shianna KV, et al. Long-range LD can confound genome scans in admixed populations. *Am J Hum Genet*. 2008; **83**(1):132-5; author reply 5-9.
11. Muthén LK, Muthén BO: Mplus User's Guide. Seventh ed. Los Angeles, CA, Muthén & Muthén; 1998-2012.
